# Journal clubs in the time of preprints

**DOI:** 10.7554/eLife.38532

**Published:** 2018-06-11

**Authors:** Prachee Avasthi, Alice Soragni, Joshua N Bembenek

**Affiliations:** 1 Department of Anatomy & Cell BiologyUniversity of Kansas Medical CenterKansas CityUnited States; 2 Department of OphthalmologyUniversity of Kansas Medical CenterKansas CityUnited States; 3 Division of Hematology-Oncology, David Geffen School of MedicineUniversity of California Los AngelesLos AngelesUnited States; 4 Department of Biochemistry, Cellular and Molecular BiologyUniversity of TennesseeKnoxvilleUnited States

**Keywords:** peer review, preprints, early-career researchers, journal clubs, open science, graduate training

## Abstract

Early-career researchers can learn about peer review by discussing preprints at journal clubs and sending feedback to the authors.

Assessing the scientific literature and providing constructive criticism is an essential skill for all scientists. Many early-career researchers learn how to review publications by discussing recent papers at journal clubs. Some graduate students and postdocs also have the opportunity to review manuscripts with their supervisors, but for many, their first contact with the formal peer review process takes place when they submit their first paper.

The past few years have seen a dramatic increase in the use of preprints in the life and biomedical sciences. Preprinting manuscripts at the author’s discretion prior to journal acceptance on a platform such as bioRxiv is changing the way and speed at which we communicate science (see Table 1 for examples of preprint servers).

**Table 1.** Examples of Preprint Servers

arXiv	A preprint server for physics, quantitative biology, statistics, mathematics and other similar fields
bioRxiv	A preprint server for the life sciences
ChemRxiv	A preprint server for chemistry
PeerJ Preprints	A preprint server for the biological, medical and environmental sciences
PsyArXiv	A preprint server for the psychological sciences
PaleorXiv	A preprint server for paleontology

One of the benefits of preprints is that authors can revise and improve their manuscript in response to feedback from readers before formal publication in a journal. Moreover, as we will discuss in this article, if journal clubs focus on preprints rather than papers published in journals, they can also be used to train students in the peer review process.

## Starting a preprint journal club

It is straightforward to convert a traditional journal club to a preprint journal club. First, an introductory presentation can detail the features of preprints to the students. Then, the fundamentals of the preprint review process can be taught through an in-depth discussion, supported by templates and resources, such as those available on the PREreview platform.

In practice, each week one student selects a preprint and presents the major findings and their analysis to the group. Meanwhile, a second student takes notes on this presentation and the subsequent discussion. The presenter and the second student then prepare a draft referee report on the preprint, which is circulated to the group for feedback. Finally, students can email the report to the corresponding authors of the preprint and post it on an online platform such as PREreview or The Winnower (see Table 2 for resources and other preprint review platforms).

**Table 2.** Examples of Preprint Review Platforms

biOverlay	An open access journal that peer reviews selected preprints and publishes a final report about them
Peer Community In	A recommendation service for preprints and published articles based on reviews by the community
preLights	A server that highlights preprints in the life sciences
PREreview	A platform for peer reviewing preprints
The Winnower	An open-access publishing platform that employs open post-publication peer review

Preprint journal clubs do not need to take place in person. Led by one of us (AS) together with David W. Sanders (Princeton University), the Phase Separation Journal Club is an example of a virtual journal club. Using the online platform Slack, it brings together about 40 members spanning the entire academic career spectrum and several time zones. Every preprinted or published paper that the group discusses has its own channel. A session starts with a bullet point write-up of the paper, which is followed by a one-hour online discussion using the chat feature. Participants can add comments later on as well. A summary of the journal club can be collated and provided as feedback to the authors.

## Preprint journal clubs: Impact and feedback

Utilizing preprints in journal clubs has many unique advantages. Students learn to critically evaluate manuscripts that have not already been peer reviewed, so there is no assumption that a panel of expert reviewers has already caught potential mistakes or improved the manuscript. By uncoupling the manuscript from a specific journal title, trainees are encouraged to review the quality of the science, with no focus on perceived impact or alignment with a specific journal scope.

In addition, the students are expected to produce a written report rather than just participate in a verbal discussion, which forces them to organize their thoughts in a coherent manner. Knowing that the authors of the preprint are going to read the report increases accountability and favors constructive rather than destructive comments. Lastly, student reports will provide additional comments to preprint authors, who currently do not experience a high level of feedback on their work (~10% on bioRxiv; Inglis and Sever, 2016).

In our experience thus far, the response from authors has been positive. One thanked the student for their comments in the acknowledgments of their paper, and others have asked students to consider joining their institution to do research. In one case, the author sent the official reviews, their response to the reviews and the revised manuscript so that the students could compare their own comments with the official reviews. Other authors have sent back point-by-point rebuttals, just as they would do in response to journal reviewers, which further extends the training potential of this exercise. Several authors also replied that they have initiated their own preprint journal clubs.

Unlike traditional journal clubs focused on published manuscripts, preprint journal clubs create the opportunity for students to provide feedback that can lead to improvement of the manuscript. Helping the authors and contributing to the scientific discourse motivates and empowers early-career researchers, building their confidence as young scientists.

Finally, when the preprint is published in a journal, the students can compare the final and original versions, and see first-hand how the manuscript has changed through the review process. This gives trainees healthy expectations about how their own manuscripts might evolve during peer review.

## Future directions for preprint evaluation

Getting credit for contributions in preprint journal club review is an important consideration for early-career researchers. Reviewing platforms such as The Winnower or PREreview already grant DOIs to reviews, which can then be added to the evaluator’s CV or ORCID profile. Journal editors are therefore able to directly evaluate the quality of the open reports provided by junior scientists, potentially leading to formal peer review opportunities.

While getting public recognition for one’s reviewing efforts may be of interest for some, it is critically important to retain the ability to maintain anonymity whenever needed or desired so that vulnerable junior and underrepresented scientists can be protected. As an example, this can be achieved on the BiOverlay platform, where an editor handles the reviews confidentially, in a way that is consistent with what happens at a journal. Similar approaches to retain anonymity must be developed and implemented for groups of students. For instance, instructors could communicate reviews to authors on behalf of students, while online journal clubs could allow participants to be anonymous if they prefer.

Beyond journal clubs, preprint reviews could be used early on in the formal education of new PhD students. The ability to scrutinize new results is an important part of the shift from trusting the textbooks to critically evaluating the literature. Just as we teach ethics and statistics to students, we should also train them in peer review practices. Students could start by using a template to review landmark papers that demonstrate exemplary scientific rigor and manuscript organization. Subsequently, they could review a recent preprint selected by the course leader. The final step is for students to choose and review a preprint, and send the report to the corresponding author after validation by the instructor. A single class has therefore the opportunity to evaluate a large number of papers.

## Conclusion

In the current reviewing system, the fate of any scientific paper is in the hands of a very small number of people, which typically includes an editor and two to four reviewers. Moreover, the burden of reviewing a large body of literature is placed on a small percentage of the scientific community (Kovanis et al., 2016). By using existing student journal clubs to provide feedback on preprints, we could increase the pool of scientists available to review manuscripts and reduce the load placed on current reviewers. In addition, preprint reviewing has the potential to tap into a greater breadth of scientific expertise, which could complement that of the assigned journal reviewers. Beyond critical review, increased participation may allow preprint authors to broaden the discussion and identify new multidisciplinary research questions. 

Also, authors have the opportunity to leverage preprint feedback to revise their manuscript prior to journal submission, which can accelerate the review process. Ultimately, preprint feedback could be shared with journal editors, who could then choose to consider it in their evaluations.

Given the benefits for authors, students, and the broader research community, proliferation of preprint journal clubs will have a profound impact on scientific communication and training. 
